# Diagenesis does not invent anything new: Precise replication of conodont structures by secondary apatite

**DOI:** 10.1038/s41598-017-01694-4

**Published:** 2017-05-09

**Authors:** Annalisa Ferretti, Daniele Malferrari, Luca Medici, Martina Savioli

**Affiliations:** 1 Department of Chemical and Geological Sciences, University of Modena and Reggio Emilia, Via Campi 103, I-41125 Modena, Italy; 2 Institute of Methodologies for Environmental Analysis, National Research Council of Italy, C.da S. Loja-Zona Industriale, I-85050 Tito Scalo (Potenza), Italy

## Abstract

Conodont elements are important archives of sea/pore water chemistry yet they often exhibit evidence of diagenetic mineral overgrowth which may be biasing measurents. We decided to investigate this phenomenon by characterising chemically and crystallographically, the original biomineral tissue and the diagenetic mineral nature of conodont elements from the Ordovician of Normandy. Diagenetic apatite crystals observed on the surface of conodont elements show distinctive large columnar, blocky or web-like microtextures. We demonstrate that these apatite neo-crystals exhibit the same chemical composition as the original fossil structure. X-ray microdiffraction has been applied herein for the first time to conodont structural investigation. Analyses of the entire conodont element surface of a variety of species have revealed the existence of a clear pattern of crystal preferred orientation. No significant difference in unit cell parameters was documented between the newly formed apatite crystals and those of the smooth conodont surfaces, thus it emerges from our research that diagenesis has strictly replicated the unit cell signature of the older crystals.

## Introduction

Conodonts, for a long time considered enigmatic, represent an extinct group of jawless vertebrates, that were the first among the group to experiment skeletal biomineralization with tooth-like elements in their feeding apparatus^1^. These elements, ranging in average size from 0.1 to 5 mm, consist of carbonate-apatite and were arranged in a bilaterally-symmetrical apparatus within the cephalic part of the animal. Relative positioning within the apparatus is denoted by letters (P, M and S). Elements, distinguished according to their morphologies as coniform, ramiform and pectiniform, are composed of two parts: a basal body of dentine-like tissue^2^ and a crown of hypermineralized tissue comparable with the enamel of other skeletonizing vertebrates^1, 3^. Both tissue layers exhibit synchronous growth by centrifugal apposition of laminae^4, 5^. These lamellae consist of francolite crystals, whose *c*-axis orientation parallels maximum growth direction.

**Figure 1:**
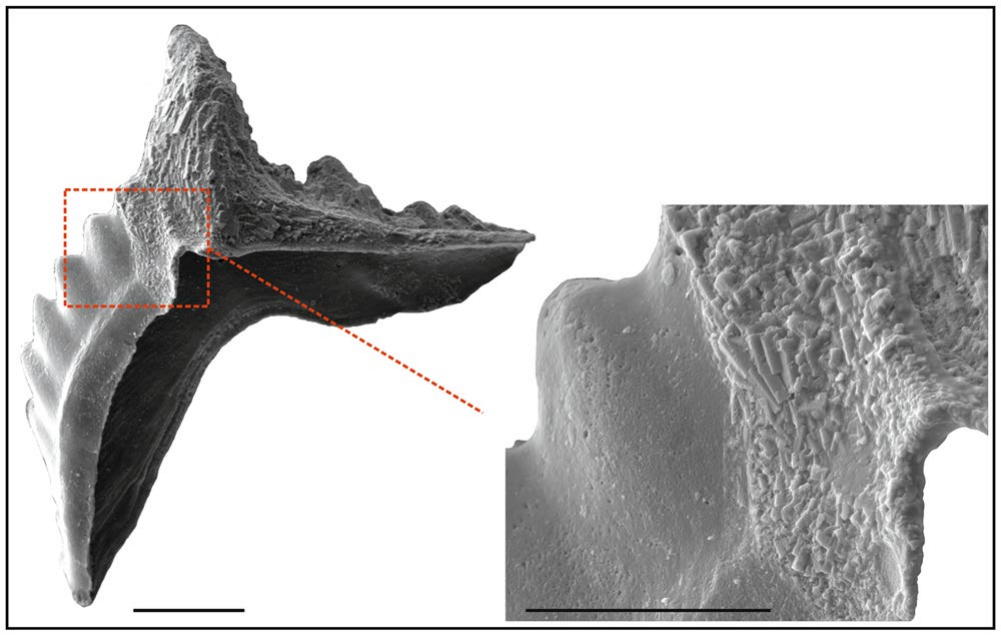
Authigenic apatite crystal overgrowth on conodonts. Scanning electron micrograph images of *Amorphognathus* sp., Pb element; specimen IPUM 28983, sample W4-2006. Note the sharp contact between the areas with microtexture and the smooth (with no crystal overgrowth) part of the element. Scale bar corresponds to 100 μm (left) and 50 μm (right enlargement).

Conodonts lived in the ancient oceans for over 300 million years from the Cambrian to the Triassic. Thanks to their rapid evolution and diversity of habitats, conodonts represent a fundamental biostratigraphical tool within carbonate depositional environments. Their phosphatic composition also facilitates significant isotope studies that provide important findings relevant in unravelling ocean geodynamics and palaeoclimates as they are important archives of sea/pore water chemistry. The presence of diagenetic mineral overgrowth has often been regarded as possibly biasing measurents. We decided to investigate this phenomenon by characterising chemically and crystallographically, the original biomineral tissue and the diagenetic mineral nature of elements from the Ordovician of Normandy. A collection of several thousand conodont elements from the Vaux Limestone exposed close to the village of Saint-Hilaire-la-Gérard has been recently referred to the Late Ordovician *A*. *ordovicicus* conodont Biozone and to the *Sagittodontina robusta*-*Scabbardella altipes* biofacies of the Mediterranean Province^6^. The conodont elements are moderately well-preserved and exhibit a CAI (Colour Alteration Index^7, 8^) of 4–5, indicating a heating of 300–400 °C. Peculiar apatite neo-crystal overgrowths on the oral surface of conodont elements were revealed during detailed scanning electron microscopy investigation. These crystals were seen to occur on elements representative of different morphologies (coniform, ramiform and pectiniform) belonging to diverse conodont species. The neo-crystal overgrowths may cover the element surface in full or only in part, with sharp boundaries observed between smooth and crystalline areas (Fig. 1). No fracturing or cleavage of the material was noted nor was indication of possible corrosion evident.

The focus of this study is to describe the above mentioned neo-crystals in terms of size, morphology, composition, geometry and spatial arrangement by integration of optical and scanning electron microscopy (SEM), environmental scanning electron microscopy coupled with chemical microanalyses (ESEM-EDX) and X-ray microdiffraction (μXRD). X-ray diffraction has been used in the past to characterize lattice parameters in apatite crystals^9–14^. μXRD in particular, applied herein to conodont structural characterisation to our knowledge for the first time, proved to be a reliable tool in describing overgrowths that otherwise cannot be resolved by the use of microscopic methods alone. In fact µXRD method allows for small volumes of material to be probed: X-rays are collimated to form a very small beam (up to 10 μm in diameter) before irradiating a sample, giving the possibility to check for local ‘micro’ environment such as defects or preferred orientations of the crystallites.

## Apatite neo-crystal overgrowth

Recrystallization and overgrowth patterns are well-documented in the literature (for a summary see ref. 15) and have been related to diagenesis, metamorphism and thermal episodes. In addition, large crystal microtexture had been referred both to early diagenesis and metamorphism of low to medium grade. Furthermore, a change from anhedral to subhedral form, alignment of long columnar crystals to primary features as well as a progressive increase in crystal size was associated with a rise in the CAI value and, consequently, with temperature^16–18^.

**Crystal overgrowth** (i.e., crystals grown from solution circulating within a metasomatic environment) differs from **recrystallization**, that is more typical of an anhydrous environment, since in the latter case, single (euhedral) superficial neo-crystals should not apparently grow paralleling the original microcrystal arrangement (e.g., following the primitive orientation of a microcrystal that is not necessarily parallel to the longitudinal axes of the denticles^19^), but should grow with an orientation driven by oriented stress imposed by regional metamorphic environment. The results from the X-ray powder diffraction analyses of the rock enclosing the conodonts under investigation do not show any significant markers of early metamorphism such as the presence of typical metamorphic minerals.

**Figure 2:**
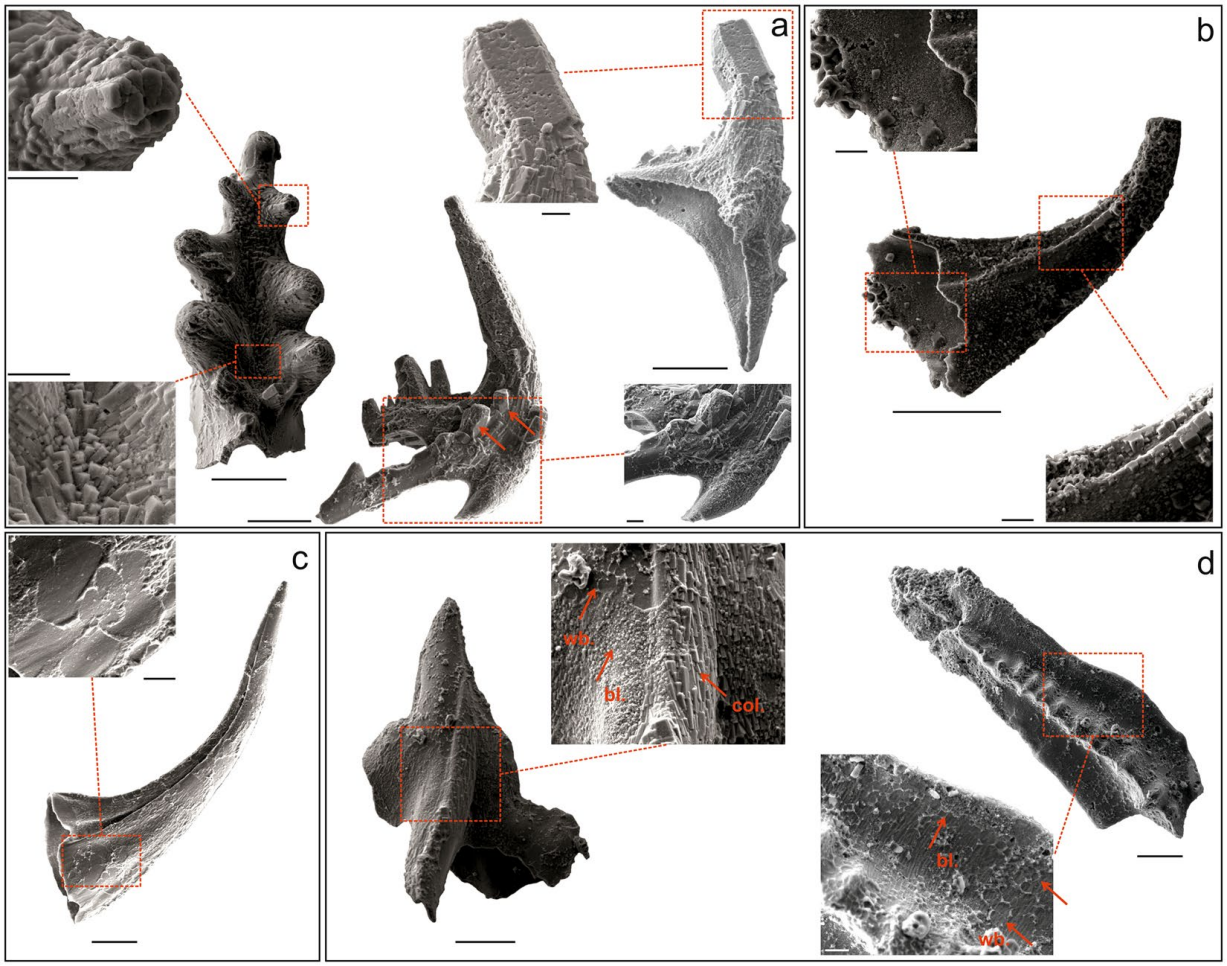
Crystal microtextures. (**a**) ESEM images of large columnar crystal microtexture in: (**a**, left) *Icriodella* sp., Pa element; specimen IPUM 28984, sample W-BK(B)-2006. Note how crystals are arranged to form rosettes to replace the original denticle (top enlargement) or configured as a sort of fan (bottom enlargement). (**a**, centre) *Amorphognathus* sp., Sd element; specimen IPUM 28985, sample W-BK(C)-2006. Red arrows indicate crystals that have replaced the original process denticles. (**a**, right) *Amorphognathus* sp., Pb element; specimen IPUM 28986, sample W-BK(C)-2006. The main denticle (cusp) has been fully replaced by a prismatic form. (**b**) ESEM images of blocky crystal microtexture in *Scabbardella altipes*; specimen IPUM 28987, sample W-BK(C)-2006. (**c**) ESEM images of web-like crystal microtexture in *Scabbardella altipes*; specimen IPUM 28988, sample W-BK(C)-2006. (**d**) ESEM images showing simultaneous presence of: (**d**, left) large columnar (col.), blocky (bl.) and web-like (wb.) crystal microtexture on the surface of *Sagittodontina robusta*, Pa element; specimen IPUM 28989, sample W1-2006; (**d**, right) blocky (bl.) and web-like (wb.) crystal microtexture on the surface of *Amorphognathus* sp., Pa element; specimen IPUM 28990, sample W1-2006. Scale bars correspond to 100 μm (conodont element) and 20 μm (enlargement).

In the samples studied apatite neo-crystals overgrown on conodont elements are seen to be arranged according to three microtextural patterns. **Large columnar crystal** microtexture (Fig. 2a) consists of long prismatic sub-isometric crystals up to 20 μm in length that are approximately aligned with the main axis of the conodont element. Columnar crystals may entirely cover the surface of the element. A single larger crystal may sometimes fully replace the process denticles (Fig. 2a, middle) or even the main cusp of the conodont element (Fig. 2a, right). **Blocky crystal** microtexture (Fig. 2b) is formed of isometric crystals up to 10 μm in length that lack a definite habit and mainly develop close to the basal cavity and along the conodont element margins. Crystals close to the element margin are independent of each other and show no order in distribution seeming like sugar-grains sprinkled over the conodont surface. Blocky crystals appear to have a normal orientation to the conodont surface when observed on the nodes and margins of the conodont element. **Web-like crystal** microtexture (Fig. 2c) consists of tiny crystals arranged as circular rims, often bordering areas with no visible crystal pattern. The resulting design is irregular suggesting a sort of net that has somehow been spread upon the conodont element surface. The latter appears to be the most common crystal microtextural arrangement which has been observed to develop also on platform elements. The three types of neo-crystal microtextural overgrowth may develop in different areas of the same conodont element (Fig. 2d, left) or be exclusive to a single conodont element.

## Composition

**Figure 3:**
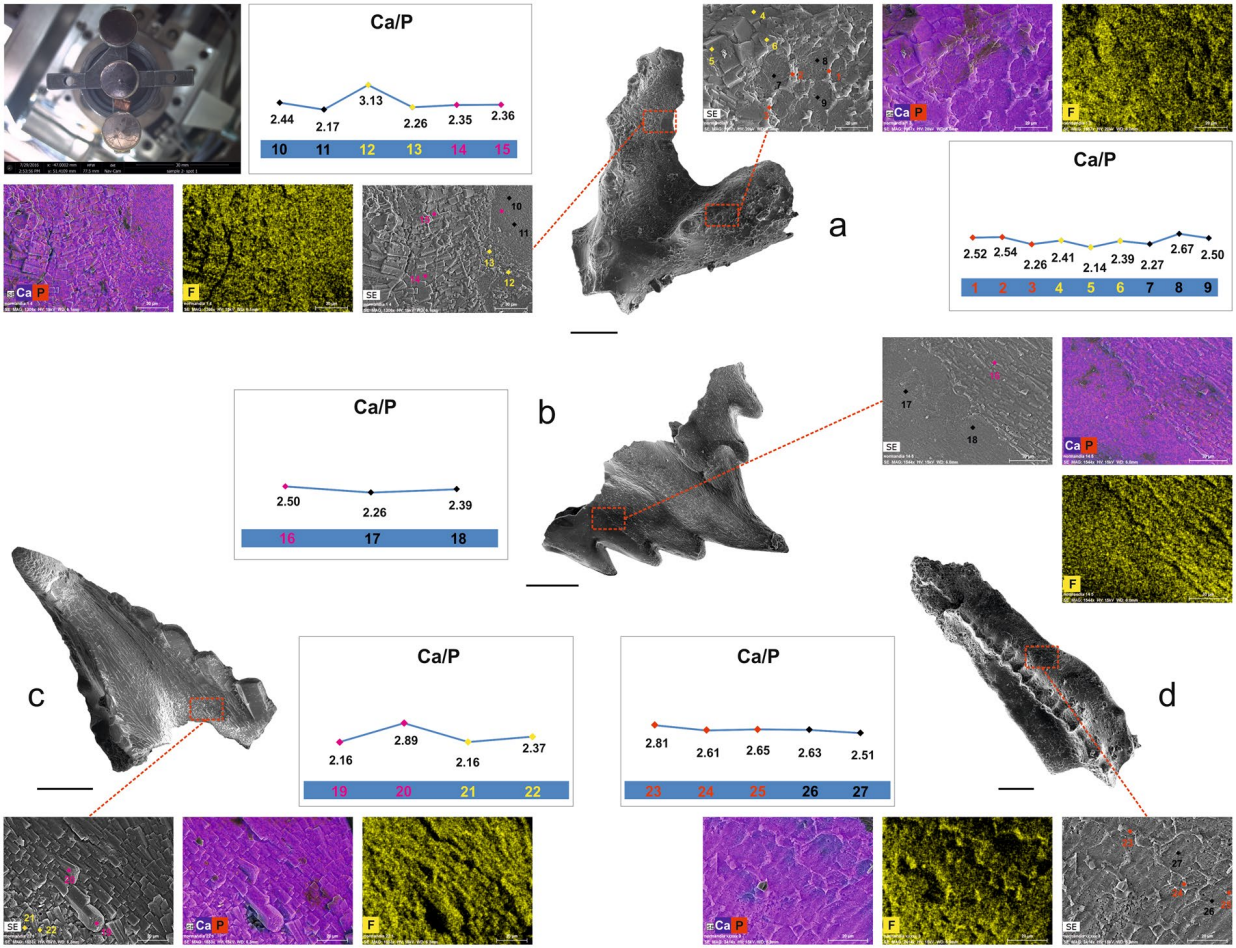
Chemical composition. ESEM image, SEM-EDS elemental maps (Ca + P and F) and Ca/P ratio. (**a**) *Sagittodontina robusta*, Pa element; specimen IPUM 28991, sample W1-2006. 1-3: web-like crystal microtexture (red spots); 4–6: blocky crystal microtexture (yellow spots); 7–9: smooth parts of the element (black spots); 10–11: smooth surface (black spots); 12–13: blocky crystal microtexture (yellow spots); 14–15: large columnar crystal microtexture (purple spots). (**b**) *Sagittodontina robusta*, Pa element; specimen IPUM 28770, sample W-BK(C)-2006. 16: large columnar crystal microtexture (purple spot); 17–18: smooth element (black spots). The marked contact between the crystalline and the smooth part of the element is notable. (**c**) *Icriodella superba*, Sa element; specimen IPUM 28761, sample W-BK(B)-2006. 19–20: large columnar crystal microtexture (purple spots); 21–22: blocky crystal microtexture (yellow spots). (**d**) *Amorphognathus* sp., Pa element; specimen IPUM 28990, sample W1-2006. 23–25: web-like crystal microtexture (red spots); 26-27: smooth surface (black spots). Scale bars correspond to 100 μm.

Environmental scanning electron microscopy coupled with microanalyses (SEM/ESEM-EDX) was applied to the entire surface of the conodont samples, thus including areas with and without neo-crystal overgrowth. Maps of major element (Ca, P, F, Mg, K, Na, Al, Si, Sr) distribution as well as Ca/P ratios do not vary significantly between the three types of neo-crystal microtextural overgrowth and the smooth areas of the conodont elements showing no overgrowth (Fig. 3). These findings support the presence of the same carbonate-fluoroapatite in all the areas analysed.

## Crystallites preferred orientation

**Figure 4:**
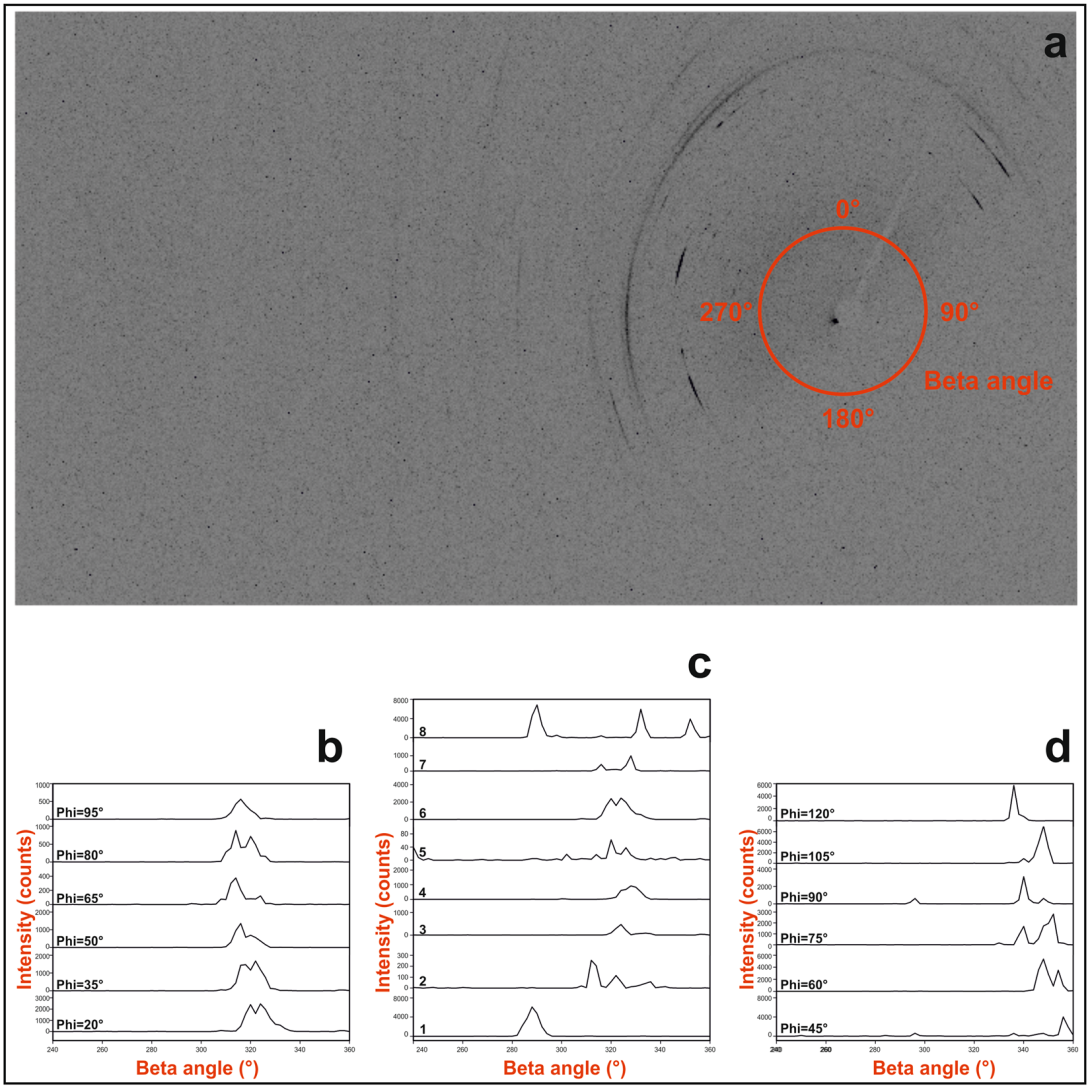
Crystallites preferred orientation. (**a**) Generic representation of Beta angle around the origin of the bidimensional image obtained by μXRD measurement. (**b**) Beta-I profiles of (300) reflection obtained by μ-XRD measurement in one point (point 6 in Fig. 6) of the surface of conodont element *Scabbardella altipes*; specimen IPUM 28988, sample W-BK(C)-2006. Omega angle fixed: 16°. (**c**) Beta-I profiles of (300) reflection obtained by μXRD in eight points of the surface of *Scabbardella altipes*; specimen IPUM 28988, sample W-BK(C)-2006. Omega angle fixed: 16°; Phi angle fixed: 20°. (**d**) Beta-I profiles of (300) reflection obtained by μXRD in one point (point 6 in Fig. 6) of the surface of conodont element *Amorphognathus* sp., Pb element; specimen IPUM 28983, sample W4-2006. Omega angle fixed: 16°. Beta angle range was selected in order to show only where the (300) reflection was revealed (240–360°).

X-ray microdiffraction was used to characterise various points of the surface of the conodont elements. The two main conodont morphologic groups of coniform elements: *Scabbardella altipes* (Henningsmoen), and ramiform elements: *Amorphognathus* sp., were investigated in this study. Peak intensities recorded at diverse angle values around the Debye rings, represented by Beta angle (Fig. 4a), were used to show the presence of preferred orientations.

Microdiffraction analyses of the *Scabbardella altipes* elements provided polycrystalline-like spectra (i.e., areas where the surface offers powder like spectra). A polycrystalline-like structure was observed and no single crystals were found on the surface of the conodont element, as proved by the bidimensional images obtained by μXRD measurements where only diffraction arcs and no diffraction spots are present^20, 21^. These data, additionally, allowed the existence of a clear pattern of preferred orientation in all the analysed points of the surface to be highlighted since some XRD reflections change in intensity (or even disappear) by varying the Phi angle^22^. Preferred orientation is also confirmed by Beta angle patterns of the (300) reflection of apatite (Fig. 4b). The (300) reflection was selected due to its intensity and distance from other reflections, features which ensure that peak intensity and position are not significantly disturbed by interference from other signals. Comparison between μXRD data measurements carried out in different points of the conodont element at the same Phi and Omega angles highlights that the polycrystalline structure does not maintain the same orientation along the surface of the conodont. This conclusion is supported by Beta angle pattern of the (300) reflection which varies both in intensity and in Beta position (Fig. 4c).

**Figure 5:**
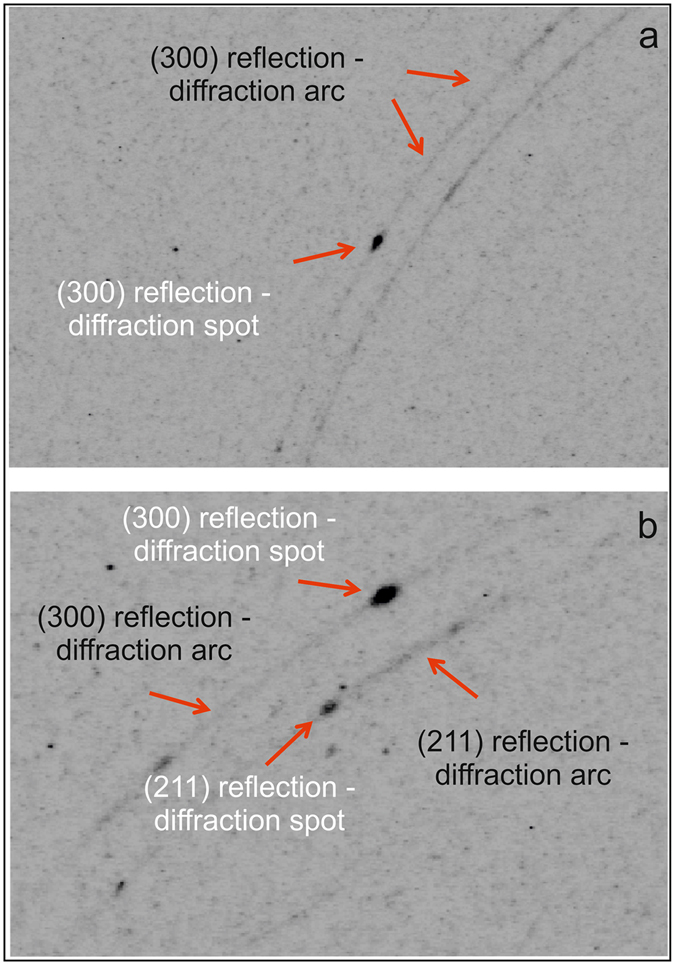
Diffraction signals. Detail of the bidimensional image obtained by μXRD measurement in one point of the surface of the conodont element. (**a**) Specimen IPUM 28983 (*Amorphognathus* sp., Pb element - point 7 in Fig. 6- centre). (**b**) Specimen IPUM 28986 (*Amorphognathus* sp., Pb element - point 5 in Fig. 6- right side).

A preferred crystallite orientation was detected also in some points of the ramiform conodont element *Amorphognathus* sp., as demonstrated by Beta angle patterns of the apatite (300) reflection (Fig. 4d). Moreover, diffraction spots in the μXRD spectra of *Amorphognathus* sp. reveal that this conodont element preserves single crystals of apatite that have grown over the polycrystalline apatite along the entire conodont body. Single crystals of apatite were detected also in another analysed ramiform element of the same conodont genus. However, in this latter case it was not possible to show the patterns of Beta angle as the strongest XRD reflections were always revealed both as diffraction arcs and spots. Figure 5a is an example of such coexistence of diffraction arc and spot signals, as no significant differences are identifiable between the (300) reflection of the polycrystalline-like diffraction arc and the (300) reflection of the single crystal-like diffraction spot (2θ = 33.09°). Comparable diffraction signals of polycrystalline apatite and single crystals of apatite were revealed also in *Amorphognathus* sp. conodont elements (Fig. 5b) for the (211) and (300) reflections (2θ = 31.81° and 33.04°, respectively).

## Unit cell parameter

**Figure 6:**
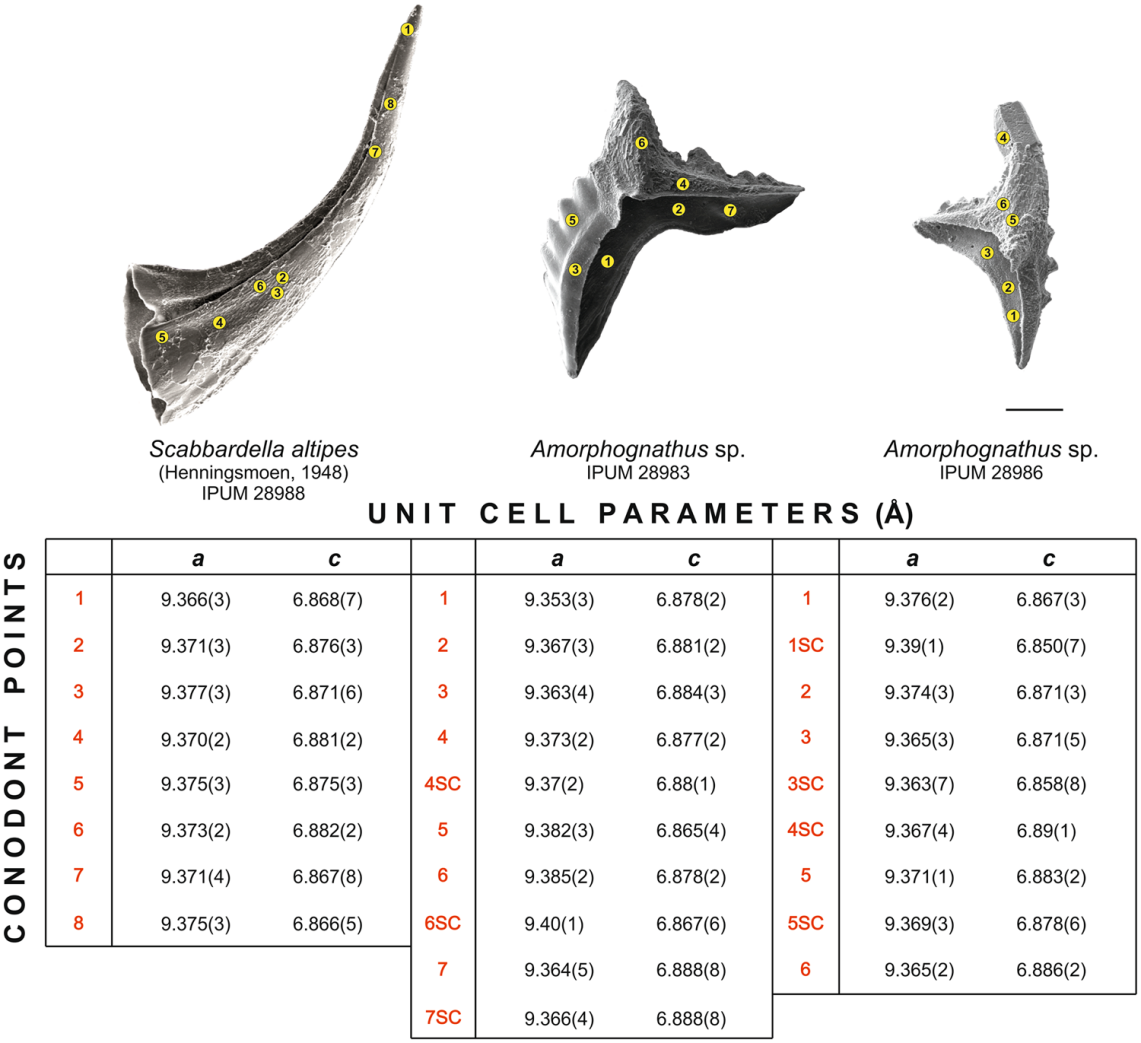
Unit cell parameters. Unit cell parameters (Å) obtained by μXRD measurements on points of the surface of the three conodonts: *Scabbardella altipes*, specimen IPUM 28988, sample W-BK(C)-2006 (left element, analysed in eight points); *Amorphognathus* sp., Pb element, specimen IPUM 28983, sample W4-2006 (middle element, analysed in seven points); *Amorphognathus* sp., Pb element, specimen IPUM 28986, sample W-BK(C)-2006 (right element, analysed in six points). Standard deviations between parentheses. When not specified, points refer to polycrystalline apatite. SC = Single crystal. Point 4 in *Amorphognathus* sp. (right element) showed large columnar crystal microtextures and did not reveal polycrystalline-like diffraction signals. High standard deviations of unit cell parameters of some single crystals derive from a limited number of reflections obtained by μXRD. Scale bar corresponds to 100 μm.

X-ray microdiffraction data also allowed calculation of the unit cell parameters. The analysed conodont element of *Scabbardella altipes* showed polycrystalline apatite all across the surface, with comparable values of *a* and slight differences among *c* cell parameters (Fig. 6, left). Web-like crystal microstructures were not distinguished with respect to the older smooth surface. Both blocky and large columnar crystal microtextures of *Amorphognathus* sp. revealed single neo-crystals of apatite perfectly overgrown on the older polycrystalline apatite. These single crystals are particularly suitable for calculation of the unit cell parameters. After six data collections for each point, measurements provided sufficient diffraction spots in order to define unit cell parameters also for the single crystals: it is worthwhile to note that *a* and *c* values did not appear to differ significantly from those calculated for the polycrystalline apatite (Fig. 6, middle-right). These values are comparable with lattice parameters detected from different taxa of Early Ordovician-late Silurian conodont elements with CAI value 1 from Estonia^14^.

## Conclusions

**Figure 7:**
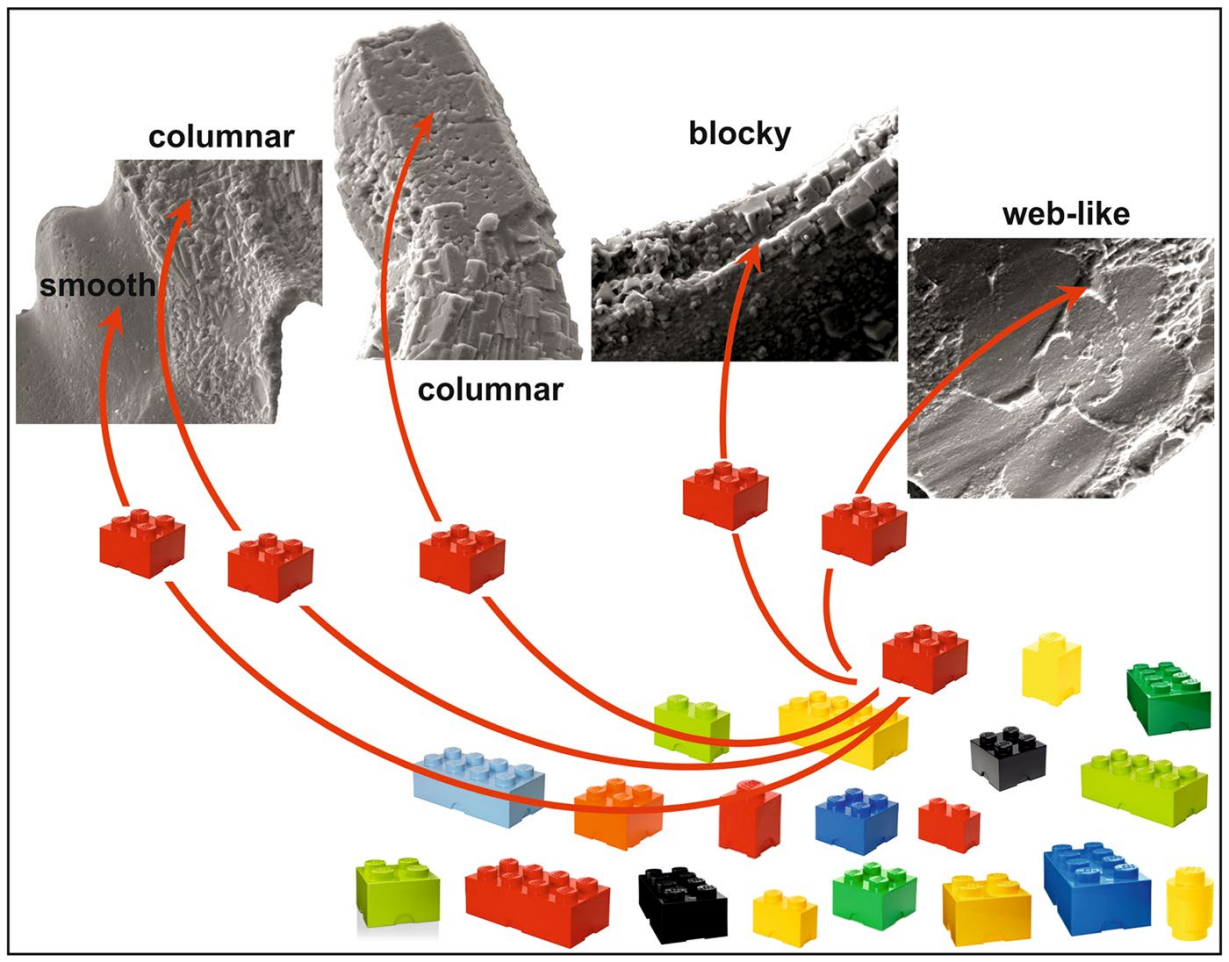
Diagenesis replicates original data: diagenetic (columnar, blocky and web-like crystal microtexture) and primary (smooth surface area) crystals share exactly the same chemical composition (red colour) and cell parameters (4-stud brick) within the large set of apatite compounds (represented by different colours and geometric bricks).

Newly formed apatite crystals were detected on the external surfaces of conodont elements from the Late Ordovician of Normandy, northern France. Neo-crystals are arranged in three crystal microtextures (large columnar, blocky and web-like) with crystals being of different size and morphology. Chemical composition remains constant and strictly replicates the composition of the original conodont element. The application for the first time of µXRD analysis to conodont structural study confirmed the existence of a preferred orientation pattern in crystallites of different lamellae. The same method facilitated definition of the unit cell parameters in the three arrangements of apatite neo-crystals observed as well as in the primary apatite crystals of the conodonts. No significant differences were noted. Apatite neo-crystals appear to have strictly replicated the chemical and structural signal of the older apatite crystals (Fig. 7).

## Methods summary

We compared over one hundred well-preserved, morphologically (coniform, ramiform, and pectiniform elements) and taxonomically different conodonts from the Late Ordovician Vaux Limestone of northern Normandy, France. Samples were processed with standard formic acid conodont technique and 100 μm–2 mm residues were concentrated by means of high-density sodium polytungstate. Heavy fractions were handpicked using a Zeiss Stemi SV 11 binocular microscope (enlargements 25–100x). Conodont elements were mounted on aluminium stubs previously covered with carbon-conductive adhesive tape. Au-coated and non-coated elements were observed using an Environmental Scanning Electron Microscope FEI ESEM-Quanta 200, equipped with an Oxford EDX INCA 300 X-ray energy dispersive spectrometer and by a Scanning Electron Microscope Nova NanoSEM FEI 450 equipped with a X**-**EDS Bruker QUANTAX-200 detector; X-ray intensities are measured by counting photons and the precision obtainable is limited by statistical error and, for major elements, the overall analytical accuracy is commonly close to ±2%. ESEM observations were performed in high and low vacuum (low vacuum brackets 1 and 0.5 Torr) with an accelerating voltage between 5 and 25 keV for imaging and between 5 and 15 keV for elemental analyses. SEM observations were in high vacuum with an accelerating voltage between 15 and 25 keV for imaging and between 15 and 25 keV for elemental analyses.

X-ray microdiffraction (μXRD) was performed on selected coniform and ramiform elements, mounted on small plane surfaces. μXRD data were acquired using a Rigaku D/MAX RAPID diffraction system, operated at 40 kV and 30 mA. This instrument is equipped with a Cu*Kα* source, curved-image-plate detector, flat graphite monochromator, variety of beam collimators, motorized stage and microscope for accurate positioning of the sample. Measurements were performed in reflection mode using a 50-μm collimator and collection times of 3 h. The μXRD data were collected as two-dimensional images and then converted into 2θ-I and Beta-I profiles using the Rigaku R-AXIS Display software. The surfaces of the conodonts were analysed in different points, and each point was analysed using certain measurements to enhance eventual preferred orientations of the crystallites of the surfaces; in order to achieve this goal, the measurements were carried out by fixing the Omega angle at 16° and by varying the Phi angle. In particular peak intensities at different angle values around the Debye rings, represented by Beta angle (Fig. 4a), were used to show the presence of preferred orientations. Unit-cell parameters were refined using UnitCell software^23^.

Material figured in the text is housed in the ‘Palaeontological Collections of the University of Modena and Reggio Emilia: under accession prefix IPUM’ at the Department of Chemical and Geological Sciences, University of Modena and Reggio Emilia, Modena, Italy.
